# Fibrochondrocyte Growth and Functionality on TiO_2_ Nanothin Films

**DOI:** 10.3390/jfb7020015

**Published:** 2016-06-15

**Authors:** Sharon Ronald, David K. Mills

**Affiliations:** 1 University of Texas Medical Branch, John Sealy Hospital, Galveston, TX 77555, USA; ronald.sharon@gmail.com; 2 Center for Biomedical Engineering and Rehabilitation Science, Louisiana Tech University, Ruston, LA 71272, USA; 3 School of Biological Sciences, Louisiana Tech University, Ruston, LA 71272, USA

**Keywords:** fibrochondrocytes, functionality, nanoassembly, substrate, titanium dioxide

## Abstract

Disorders affecting the temporomandibular joint (TMJ) are a long-standing health concern. TMJ disorders (TMJD) are often associated with an internal disc derangement accompanied by a suite of symptoms including joint noises, jaw dysfunction, and severe pain. The severity of patient symptoms and their reoccurrence can be alleviated to some extent with conservative therapy; however, refractory cases often require surgery that has shown only limited success. Bioengineered scaffolds with cell supportive surfaces an d nanoarchitectures that mimic TMJ tissue structure may offer an alternative treatment modality. In this study, titanium dioxide (TiO_2_) nanothin films, fabricated by layer-by-layer assembly, were examined as means for creating such a scaffold. The viability and growth of TMJ discal fibrochondrocytes (FCs) were assessed through MTT and DNA assays and total protein content over a 14-day experimental period. ELISA was also used to measure expression of types I and II collagen, decorin and aggrecan. Quantitative analyses demonstrated that FCs synthesized characteristic discal matrix proteins, with an increased production of type I collagen and decorin as opposed to collagen type II and aggrecan. A stimulatory effect on discal FC proliferation and extracellular matrix (ECM) expression with thicker nanofilms was also observed. The cumulative results suggest that TiO_2_ nanofilms may have potential as a TMJ scaffolding material.

## 1. Introduction

The temporomandibular joint (TMJ) disc is an elastic fibrocartilagenous tissue located between the head of the mandibular condyle and the squamous portion of the temporal bone [1,2,3]. The disc functions as an integral component of the TMJ and aids in joint lubrication, cushions and absorbs shock, and promotes smoothness of movement between the articulating surfaces [3,4,5]. Fibrochondrocytes and fibroblasts are the two cell types found within the disc [2,4,6,7]. Fibrochondrocytes predominate within the disc itself, and fibroblasts are more numerous in the attachment tissues and the junction of the disc with surrounding tissues [6,7,8]. The disc consists of an extensive extracellular matrix (ECM) with type I collagenous fiber bundles predominating [2,4,5,6]. Interspersed between the collagenous fiber bundles are elastic and type II collagen fibers and the proteoglycans aggrecan and decorin [1,2,7,8,9,10,11].

Soft tissue remodeling or degenerative changes due to derangements of the TMJ disc can impair normal joint function and cause acute or chronic pain symptoms. Surgeries, including discectomy, eminectomy, condylotomy or, procedures involving repositioning of the disc such as disc/condylar plication, discal suturing or a combination of different surgical procedures, is usually suggested as a permanent treatment option in alleviating TMJ disorders’ (TMJD) symptoms [12,13,14]. However, such treatment modalities often involve significant post-surgical complications and have demonstrated limited successes in treating TMJD often requiring revision surgery [5,10,12]. As TMJD patients have limited treatment options [15,16], the development of a functional bioengineered TMJ repair tissue becomes a critical need [17,18]. The essence of bioengineering discal tissues lies in the choice of a viable biomaterial that supports cell adhesion, growth and functionality leading to the formation of a new tissue [19,20,21]. The emphasis in the bioengineering of many soft and hard tissues has been placed on scaffolds with a modified surface that enhance cellular interactions with the scaffold’s surface [22,23,24,25]. Diverse surface modification techniques, thermal deposition, chemical self-assembly, microfabrication, physisorption, plasma spraying, particle blasting, and acid etching, have been developed [26,27]. However, these techniques pose some problems including intrinsic defects, uneven deposition and poor film adhesion, instability, microcracks and manufacturing difficulties [26,27,28].

One method that that may hold potential for TMJ bioengineering is layer-by-layer (LBL) nanoassembly. LBL nanoassembly is based on the principle of electrostatic attraction between oppositely charged particles [28,29]. LBL nanoassembly is a versatile technique and offers promise as a potential fabrication method for modifying scaffolds and implant surfaces. LBL scaffolds retain the biological activity of biomolecules adsorbed/embedded within the film and facilitate increased cell phenotype retention, cell growth and directed differentiation [30,31,32,33,34]. Also, LBL technique offers manufacturing feasibility in modifying specific features of the scaffolds such as stiffness, topography, and surface chemistry, suitable for its use as an implant scaffold or as a drug delivery system [22,28,29].

The current study assessed the potential of LBL assembled TiO_2_ nanofilms as a potential scaffold or surface coating material for repairing damaged TMJ tissues. Cell viability, growth and functionality of TMJ fibrochondrocytes on TiO_2_ nanosubstrates were studied. The effects of increasing TiO_2_ bilayers on FC response were also examined. Fibrochondrocytes proliferated and produced an abundant ECM on all nanofilms with a marked increase observed in FC functionality on thicker TiO_2_ nanofilms. This study suggests TiO_2_ nanofilms may hold potential as a method for producing materials for the repair of damaged or diseased TMJ tissues.

## 2. Results

### 2.1. Cytotoxicity Assays

**Figure 1:**
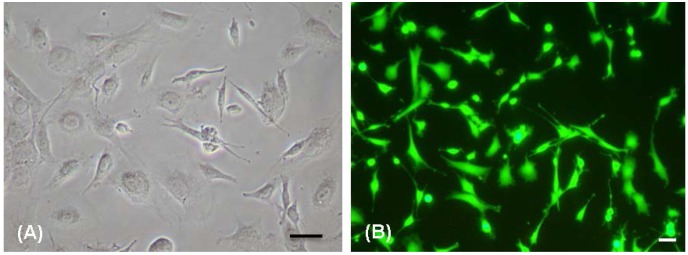

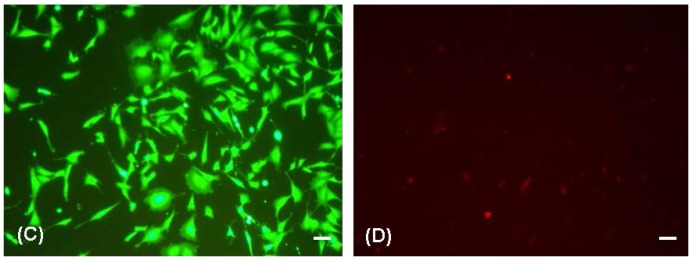
Cell culture on 5 and 10 layered TiO_2_ nanosubstrates at culture days 7 and 14. Scale bar = 10 μm. (**A**) Phase contrast microscopic image indicates FC adherence and proliferation at culture day 7on 5 layered TiO_2_ nanosubstrate; Typical FC morphology (oval and polygonal) observed on the nanosubstrate (**B**–**D**) indicate Live Dead Assay images. The green fluorescent dye (Live Dye™) indicates viable FC cell culture and the red fluorescent (propidium iodide) indicates dead cells. (**B**) Indicates FCs at culture day 7 on 5 layered TiO_2_ nanosubstrate. (**C**) And (**D**) indicates FCs at culture day 14 10 layered TiO_2_ nanosubstrate.

The current study aimed to quantitatively analyze FC proliferation and functionality with increased TiO_2_ surface coatings. Prior to proceeding to the quantitative assays, FC adherence and cytocompatibility on TiO_2_ nanothin films with increasing thickness was tested. Phase contrast observations showed TMJ fibrochondrocytes attached easily to the TiO_2_ surfaces and spread across the nanosubstrates as seen in Figure 1A. Many cells displayed the oval or polygonal cell morphology of typical fibrochondrocytes. Live Dead assays confirmed these observations and showed that the cells on the substrates thrived and increased in cell density over the experimental period (Figure 1B–D) with the expected result that TiO_2_ nanosubstrates provided a cytocompatible, cell adherent surface supporting FC phenotype stability.

### 2.2. Proliferation Assays

#### 2.2.1. MTT Proliferation Assay

**Figure 2:**
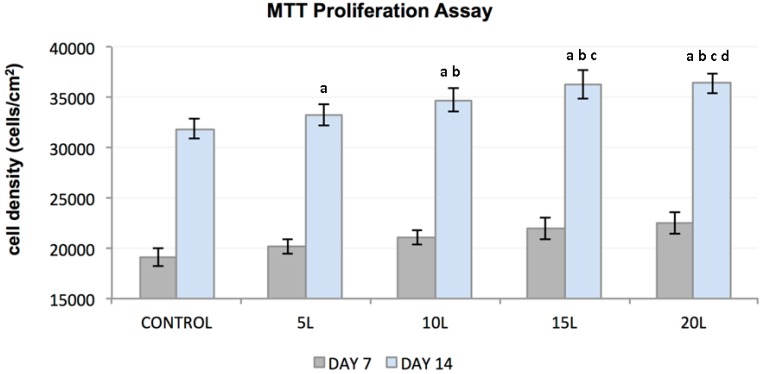
Plot represents FC cell density (*y*-axis) as a function of control substrates and increasing nanolayers of TiO₂ nanosubstrates (*x*-axis) at 7 and 14 culture days. All data are shown as mean ± standard deviation, with a sample size of 6 for all substrates. Substrates represented by letters different (a, b, c, d) are considered statistically significant (*p* < 0.05), using two-way analysis of variance. “a” indicates nanosubstrates that show statistically significant cell numbers w.r.t control substrates. “b”, “c” and “d” indicates nanosubstrates that show statistically significant cell numbers w.r.t 5 L, 10 L and 15 L nanosubstrates respectively.

An increased rate of FC proliferation was observed with increased days in culture and for all the substrates (Figure 2). Enhanced proliferation was seen on culture day 14 compared to culture day 7 for all substrates. An increased number of proliferating cells occurred on TiO_2_ nanosubstrates as compared to control substrates. An increase in the number of TiO_2_ nanolayers deposited on the LBL scaffolds was observed to be a potential factor in promoting cellular growth. Statistical analyses conducted on the MTT results demonstrated that FC proliferation was statistically significant (*p* < 0.05) with an increase in culture days and with increasing nanothin film thickness.

#### 2.2.2. DNA Quantitation Assay

**Figure 3:**
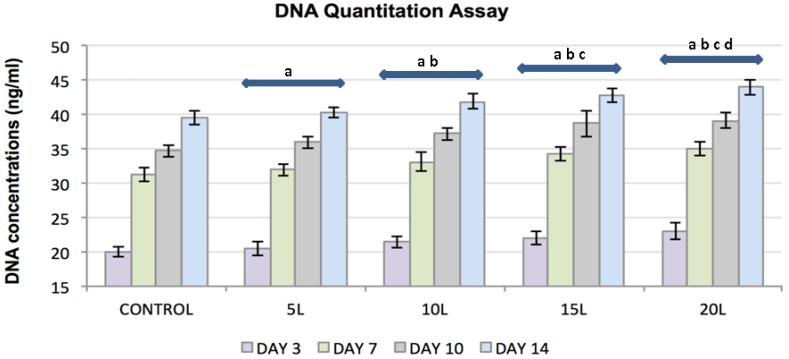
Plot represents DNA concentration of FC lysate samples (*y*-axis) as a function of control substrates and increasing nanolayers of TiO₂ nanosubstrates (*x*-axis) at 3, 7, 10 and 14 culture days. All data are shown as mean ± standard deviation, with a sample size of 6 for all substrates. Substrates represented by letters different (a, b, c, d) are considered statistically significant (*p* < 0.05), using two-way analysis of variance. “a” indicates nanosubstrates that show statistically significant cell numbers w.r.t control substrates. “b”, “c” and “d” indicates nanosubstrates that show statistically significant cell numbers w.r.t 5 L, 10 L and 15 L nanosubstrates respectively.

The DNA quantitation assay substantiated the results obtained from the MTT proliferation assay (Figure 3a,b). FC response was evaluated for a culture period of 3, 7, 10 and 14 days for an accurate assessment of FC proliferative rate. FC proliferation increased over a period of 14 days and with increasing TiO_2_ nanofilms, although enhanced proliferation on the nanosubstrates was not observed from the control substrates. Statistical analyses on the DNA Quantitation results corroborated with the MTT results demonstrating statistically significant (*p* < 0.05) FC proliferation with increasing culture days, day 14 as compared to day 7, and with increasing nanolayers.

### 2.3. Protein Assays

#### 2.3.1. Bradford Assay

**Figure 4:**
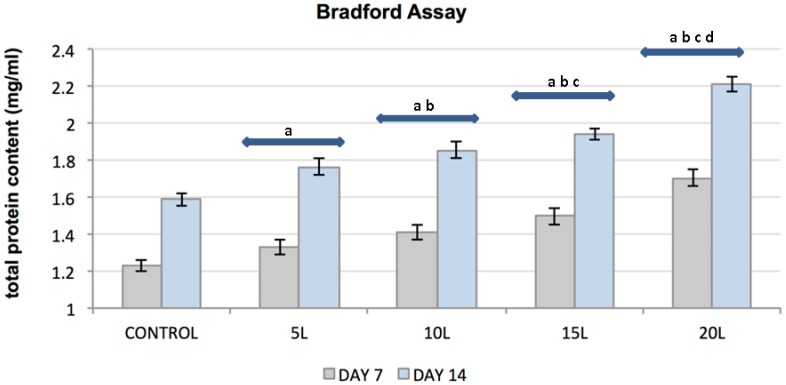
Plot represents FC total protein concentration (*y*-axis) as a function of control substrates and increasing nanolayers of TiO₂ nanosubstrates (*x*-axis) at 3, 7, 10 and 14 culture days. All data are shown as mean ± standard deviation, with a sample size of 6 for all substrates. Substrates represented by letters different (a, b, c, d) are considered statistically significant (*p* < 0.05), using two-way analysis of variance. “a” indicates nanosubstrates that show statistically significant cell numbers w.r.t control substrates. “b”, “c” and “d” indicates nanosubstrates that show statistically significant cell numbers w.r.t 5 L, 10 L and 15 L nanosubstrates respectively.

The total protein produced by fibrochondrocytes (FCs) on TiO_2_ nanosubstrates and control substrates increased with the number of culture days and with an increase in the number of TiO_2_ nanolayers. A substantial increase in FC functionality was observed with an increased in the number of layers of TiO_2_ nanosubstrates as compared to control substrates (Figure 4). Statistical analyses on the Bradford assay indicated statistically significant (*p* < 0.05) FC functionality with an increase in culture days and with increasing nanothin film thickness.

#### 2.3.2. ELISA

**Figure 5:**
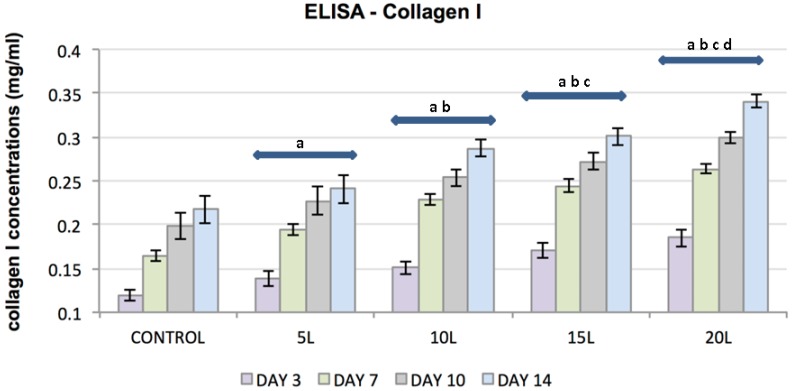
Plot represents FC collagen I concentration (*y*-axis) as a function of control substrates and increasing nanolayers of TiO₂ nanosubstrates (*x*-axis) at 3, 7, 10 and 14 culture days. All data are shown as mean ± standard deviation, with a sample size of 6 for all substrates. Substrates represented by letters different (a, b, c, d) are considered statistically significant (*p* < 0.05), using two-way analysis of variance. “a” indicates nanosubstrates that show statistically significant cell numbers w.r.t control substrates. “b”, “c” and “d” indicates nanosubstrates that show statistically significant cell numbers w.r.t 5 L, 10 L and 15 L nanosubstrates respectively.

**Figure 6:**
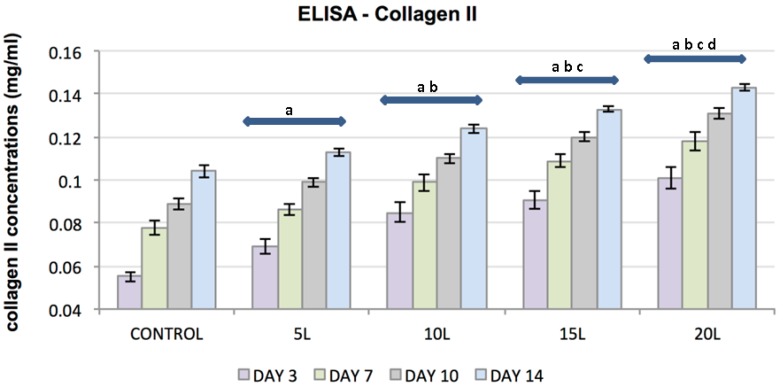
Plot represents FC collagen II concentration (*y*-axis) as a function of control substrates and increasing nanolayers of TiO₂ nanosubstrates (*x*-axis) at 3, 7, 10 and 14 culture days. All data are shown as mean ± standard deviation, with a sample size of 6 for all substrates. Substrates represented by letters different (a, b, c, d) are considered statistically significant (*p* < 0.05), using two-way analysis of variance. “a” indicates nanosubstrates that show statistically significant cell numbers w.r.t control substrates. “b”, “c” and “d” indicates nanosubstrates that show statistically significant cell numbers w.r.t 5 L, 10 L and 15 L nanosubstrates respectively.

**Figure 7:**
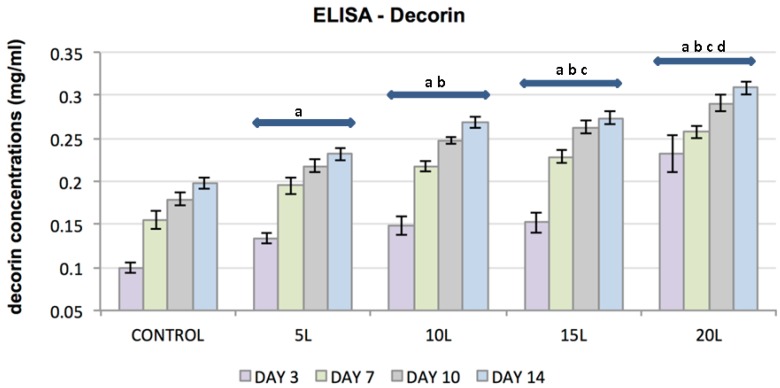
Plot represents FC decorin concentration (*y*-axis) as a function of control substrates and increasing nanolayers of TiO₂ nanosubstrates (*x*-axis) at 3, 7, 10 and 14 culture days. All data are shown as mean ± standard deviation, with a sample size of 6 for all substrates. Substrates represented by letters different (a, b, c, d) are considered statistically significant (*p* < 0.05), using two-way analysis of variance. “a” indicates nanosubstrates that show statistically significant cell numbers w.r.t control substrates. “b”, “c” and “d” indicates nanosubstrates that show statistically significant cell numbers w.r.t 5 L, 10 L and 15 L nanosubstrates respectively.

**Figure 8:**
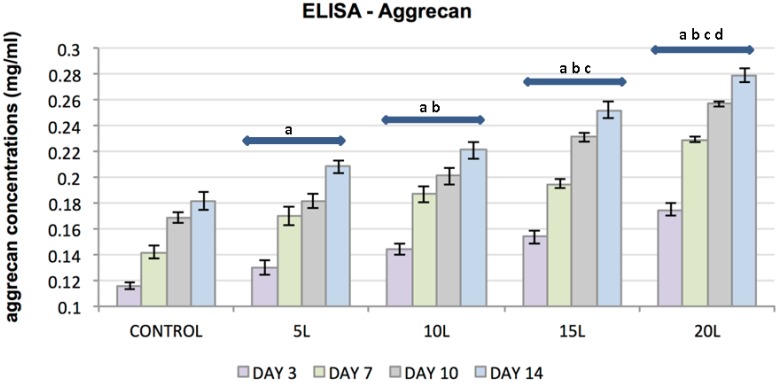
Plot represents FC aggrecan concentration (*y*-axis) as a function of control substrates and increasing nanolayers of TiO₂ nanosubstrates (*x*-axis) at 3, 7, 10 and 14 culture days. All data are shown as mean ± standard deviation, with a sample size of 6 for all substrates. Substrates represented by letters different (a, b, c, d) are considered statistically significant (*p* < 0.05), using two-way analysis of variance. “a” indicates nanosubstrates that show statistically significant cell numbers w.r.t control substrates. “b”, “c” and “d” indicates nanosubstrates that show statistically significant cell numbers w.r.t 5 L, 10 L and 15 L nanosubstrates respectively.

ELISA was used to evaluate the effect of TiO_2_ nanosubstrates and its increasing nanolayers on ECM protein expression. The increase in protein content was assessed at culture days 3, 7, 10 and 14. TiO_2_ nanosubstrates and the control substrates supported the production of collagen types I and II and the proteoglycans decorin and aggrecan with increased protein production over the 14-day experimental period (Figure 5, Figure 6, Figure 7 and Figure 8). Protein expression was substantially pronounced with increasing layers of TiO_2_ nanosubstrates than control substrates. A prominent increase in collagen I was observed on the TiO_2_ nanosubstrates followed by an increased production of decorin, aggrecan and collagen II. Statistical analysis indicated statistically significant (*p* < 0.05) ECM expression of the assessed proteins with increasing culture days and an increase in nanothin film thickness. With thicker nanofilms being a factor in promoting enhanced FC functionality, TiO_2_ nanosubstrates could eventually aid in creating a potential scaffold mimicking the TMJ environment.

### 2.4. Statistical Significance

Two-way analysis of variance on the quantitative assays indicated higher statistical significance of FC proliferation and FC ECM markers, observed with increasing culture days and increased nanothin film thickness. Significant differences in the *F* value and *F*_crit_ value, with *F* > *F*_crit_, was observed for culture days and the substrates employed, for the quantitative assays. Statistical analysis of the results suggests that increased TiO_2_ nanothin films may be promoting an increase in FC proliferation resulting in enhanced FC functionality and an increase in specific markers of TMJ disc protein expression.

## 3. Discussion

The current study describes the first successful attempt to grow TMJ discal fibrochondrocytes on TiO_2_ nanoparticle substrates prepared through LBL nanoassembly. Increasing TiO_2_ nanofilm thickness was examined as a means for enhancing cell adhesion and functionality. Increase in cell proliferation with an increase in TiO_2_ film thickness was observed, along with a stimulatory effect on protein synthesis. Enhanced protein synthesis on thicker TiO_2_ nanosubstrates was evidenced by an increased expression in discal specific ECM proteins including collagen types I and II, aggrecan, and decorin. Synthesis of a fibrous matrix (type I collagen and decorin) predominated over a cartilaginous matrix (type II collagen and aggrecan), a pattern comparable to the native TMJ disc ECM where a fibrous matrix dominates over a localized cartilaginous matrix [1,2,4,8,17]. A stimulus towards FC proliferation may be seen as a positive outcome in that an increase in film thickness promoted cell growth but also provided a means for increased ECM synthesis.

LBL constructed TiO_2_ nanofilms has shown prior success as a surface modification technique for bone implants [35,36]. Increasing TiO_2_ film thickness was also linked to an enhanced osteoblastic and mesenchymal stem cell response [22,31,36,37]. Notably, in their conclusions, the authors pointed to an increase in TiO_2_ film thickness and linked this with an increase in cell adhesion, proliferation, and matrix mineralization. A transitory shift from increased osteoblast proliferation to early matrix mineralization with thicker nanofilms was observed in a companion study, again demonstrating the importance of thicker TiO_2_ nanofilms and cell functionality [35,37].

While our results are encouraging several factors still need to be investigated for the potential of LBL fabricated TiO_2_ nanothin films for TMJ tissue repair, or regeneration may be recognized. What physical properties (surface roughness, hydrophilicity, porosity, or stiffness) of the TiO_2_ nanosubstrates promoted protein synthesis over cell proliferation? Cell source and cell density have been widely recognized as a major challenge in TMJ bioengineering [17,18,19,38]. TMJ disc cells, articular chondrocytes, and costal chondrocytes have been studied extensively for use in bioengineering TMJ tissues [38,39]. However, many investigators have cited the lack of clinical translatability of these cells for engineering new discal and mandibular condyle tissues [17,18,19,38,39]. Control over phenotype was also an issue identified in several studies using various types of TMJ cells. In many discal constructs generated, the seeded cells developed a more chondrocytic phenotype, with a rounded morphology, and positive staining for aggrecan and type II collagen and an overall matrix with more hyaline-like cartilage characteristics [39,40,41,42,43]. Recently, costal chondrocytes have showed promise as a cell source [44,45,46]. It was demonstrated that costal chondrocyte scaffold-less constructs could be generated with cellularity and a glycosaminoglycan content significantly greater that the disc-cell constructs, with retention of overall size and shape [44,45]. Stem cells have also emerged as a promising cell source for mandibular condyle tissue regeneration, although more investigation is required before their medical potential can be fully known [46,47,48].

This study and others have shown that TiO_2_ nanofilms support fibroblast, chondroblast, osteoblast, and mesenchymal stem cell grow and permit synthesis of an appropriate tissue matrix [22,31,32,33,35,36,37,38]. TiO_2_ films can be formed on many surfaces and polymers including poly(lactide), polycaprolactone, and poly(D,L lactic acid) (PDLLA) [49,50,51] and modulated for the desired surface characteristics. Gerhardt *et al.* (2007), for example, used TiO_2_ nanoparticles to create a nanometer surface topography and to enhance roughness on PDLLA for bone tissue engineering [52].

Further control over matrix production by FCs might be obtained with embedded drugs, growth factors or proteins used as biological signaling agents [30,32,40,41]. The role of an instructional agent such as BMP-2 or TGF-β or the synergistic effect of different instructional agents at their effective dosage may be a means to support a regulated increase in ECM synthesis on TiO_2_ nanofilms. In addition, the potential additive role of biomechanical stimuli and its effects on nanofilm integrity and longevity need to be investigated. Accordingly, TiO_2_ LBL nanofilms could be custom tailored to the biological requirements of a range of TMJ tissues including bone, cartilage, ligaments, and tendon according to the cell and tissue requirements of the affected area.

The past successes of LBL fabricated TiO_2_ nanothin films indicate the potential of this novel technique in contributing critical features to a scaffolding material for TMJ disc reconstruction or repair. Although finding an ideal TMJ discal scaffold for repair or regeneration remains a critical challenge, the current study on TiO_2_ nanofilms, suggests it may have potential as a TMJ scaffolding material.

## 4. Materials and Methods

### 4.1. Fibrochondrocyte Isolation and Maintenance

TMJ discs extracted from sacrificed bovine heads (CKC Farms, Pitkin, LA, USA) were employed in isolating FCs. Complete Dulbecco’s Modified Eagle Medium (DMEM) (Biosource, Rockville, MD, USA) was used in culturing the obtained cells and, consisted 500 mL of DMEM supplemented with 10% FBS (Fetal Bovine Serum) (Biosource, Rockville, MD, USA), 10 μg/mL ascorbic acid (Sigma, St. Louis, MO, USA), and 5% penicillin-streptomycin-fungizone (PenStrep). Prior to tissue dissociation, the discs were sterilized with a thorough rinse in 3X Hank’s Balanced Salt Solution (HBSS) (Biosource, Rockville, MD, USA) supplemented with 1X PenStrep (Biosource, Rockville, MD, USA), followed with a 24-hour incubation in complete DMEM to which 5X PenStrep was added. Enzymatic digestion of the disc with collagenase (Biosource, Rockville, MD, USA) and pronase (Biosource, Rockville, MD, USA) and, mechanical stirring of the tissue digest released FCs. FCs were then plated in 60 mm tissue culture plastic (TCP) (Biosource, Rockville, MD, USA) at an appropriate density. The cells were maintained in complete DMEM in a humidified environment at 37 °C, 5% CO_2_ and 95% air. Until a required confluence was obtained, the media was replaced every 24 h after initial cellular attachment. Fibrochondrocytes were passaged twice to obtain an appropriate density of cells for the quantitative analyses.

### 4.2. TiO_2_ LBL Nanothin Film Formation

An automated robotic dipping machine (manufactured by Riegler and Kirstein Gmbh, Potsdam, Germany), was used in the preparation of nanoscale substrates for mass production and uniformity in film deposition. Sterile, hydrophilic glass substrates were used as control substrates and, for the deposition of nanothin films via the LBL fabrication technique. Precleaned glass substrates were sterilized with ultrasonication in 50% isopropanol, overnight immersion in 75% ethanol solution, thorough rinse with DI followed by drying the glass substrates with a nitrogen gas stream. The nanometer- thick thin films were formed on sterile glass substrates via the conventional dipping technique. Nanosubstrates consisted of 3 alternating layers of Poly (styrene sulfonate) sodium salt (PSS, MW ~ 70 kDa, Sigma, Polyanion) and Poly (dimethyldiallyl ammonium chloride) (PDDA, MW ~ 200 kDa, Sigma, Polycation), with a thickness of about 10nm as precursor layers, onto which active layers were deposited for uniformity and better adhesion. Cationic TiO_2_ nanoparticles (diameter ~21 nm, Aeroxide TiO_2_, P25 Degussa AG, Mari, Germany) used in alternation with anionic PSS formed the active layers, with TiO_2_ being the terminating layer in all cases. (Complete details on TiO_2_ nanofilms assembly and component preparation are mentioned in reference [31]). TiO_2_ nanosubstrates (5,10,15 and 20-layered) were prepared and evaluated for FC response.

### 4.3. Fibrochondrocyte Seeding on TiO_2_ Nanosubstrates and Control Substrates

Nanosubstrates and control substrates were sterilized with its’ overnight immersion in 75% alcohol and a thorough rinse in HBSS, before FC seeding was ensued. FC’s at a density of about 5000 cells/cm^2^ were then plated and the seeded substrates were placed in 24 well plates containing complete DMEM and, were maintained in a humidified environment at 37 °C, 5% CO_2_ and 95% air for cellular attachment and confluence. Quantitative analyses and viability studies were then performed on FC seeded substrates and FC response was evaluated at different culture period.

### 4.4. Live Dead Cytotoxicity Assay

The viability studies were performed using a Live/Dead Cell cytotoxicity assay (Biovision, Milpitas, CA, USA). The kit contains Live-Dye™ a cell-permeable green fluorescent dye (*E*_x_/*E*_m_ = 488/518 nm), to stain live cells in green, and propidium iodide (PI), a cell non-permeable red fluorescent dye (*E*_x_/*E*_m_ = 488/615), to stain dead cells in yellow-red. For viability studies, staining solution was prepared by mixing 1 μL of solution A (1 mM Live-Dye) and 1 μL of solution B (2.5 mg/mL PI) to 1 mL of Staining Buffer. The seeded substrates were washed twice with HBSS and then 150 μL of staining solution was added to each substrate. These substrates were then incubated at 37 °C for 15 min. Slides were assessed for cell viability and observed on day 7, 14, and 21 using an Olympus BX51 epifluorescent microscope and representative images were captured using a Nikon digital camera.

### 4.5. Proliferation Assays

#### 4.5.1. MTT Proliferation Assay

The MTT assay determined FC proliferative rate on different layered (5,10,15 and 20) TiO_2_ nanosubstrates and control substrates at 7 and 14 culture days. The principle of the assay is based on the reduction of yellow tetrazolium MTT, 3-[4,5-dimethylthiazol-2-yl]-2,5-diphenyl-tetrazolium bromide, to purple formazan crystals that indicated cellular mitochondrial activity and thus proliferating cell densities in a given culture. FC seeded substrates were rinsed with HBSS free of phenol red and incubated with MTT working solution, at the end of the culture period. The substrates were incubated for about 4 h in a CO_2_ incubator at 37 °C, at a volume that was 10% of the volume of media (complete DMEM). Interaction of mitochondrial dehydrogenase enzyme present in the metabolically active viable cells with the yellow MTT generated reducing equivalents NADH and NADPH that yielded intracellular purple formazan crystals on the surface of the substrates at the end of the incubation period. The crystals were then solubilized using acidified isopropanol and quantified for FC proliferation. Spectrophotometric quantification of proliferating FC’s on the substrates’ surface (at a wavelength of 570 nm and a reference wavelength of 690 nm) and, the linear relationship established between FC densities and the corresponding absorbance measurements (graph not given) enabled the accurate quantification of the proliferating FC densities. Six samples were considered for each type of substrate and for all assays to obtain accurate readings.

#### 4.5.2. DNA Quantitation Assay

DNA assay was used to test the accuracy and reliability of the MTT results. In our hands, the MTT assay often provides results that may be incorrectly interpreted. The assay assessed the proliferative activity of FCs on different layered (5, 10, 15 and 20) TiO_2_ nanosubstrates and control substrates at culture period of 3, 7, 10 and 14 days The principle of the assay is based on detecting the amount of dsDNA produced during the S phase with an ultrasensitive fluorescent nucleic acid stain, Picogreen reagent, indicating the number of dividing cells during mitosis. A standard DNA curve (graph not given) established between different FC densities and fluorescing dsDNA samples enabled to obtain the number of proliferating cells from the fluorescing samples at an excitation wavelength of 485 nm and emission wavelength of 535 nm. Six samples were considered for each type of substrate and for all assays to obtain accurate readings.

### 4.6. Protein Assays

#### 4.6.1. Bradford Assay

Bradford assay quantified the total protein content produced by different layered (5, 10, 15 and 20) TiO_2_ nanosubstrates and control substrates at 7 and 14 culture days. The assay essentially made use of Bradford dye, Coomasie^@^R Brilliant Blue G-250, which exhibited differential color change on binding to various concentrations of protein present in the given samples. Absorbance from the dye-binding calorimetric reaction was measured spectrophotmetrically at a wavelength of 595 nm and, the total protein content was quantified from the standard protein curve established between different concentrations of Bovine Serum Albumin (BSA) and the corresponding absorbance measurements at different BSA concentrations.

#### 4.6.2. ELISA

The biosynthetic activity of TiO_2_ nanosubstrates and control substrates were realized with the expression of type I and type II collagen, aggrecan and decorin, the significant TMJ ECM phenotype markers. ELISA was used to detect the specific ECM protein production on different layered (5, 10, 15 and 20) TiO_2_ nanosubstrates and control substrates at culture period of 3, 7, 10 and 14 days. A suite of anti-cartilage matrix monoclonal antibodies was used in the calorimetric detection of the antigens of interest. The primary antibodies used for collagen I M-38), collagen II (CIICI) and decorin (DS1) were purchased from the Developmental Hybridoma Labs, University of Iowa, Iowa City, IA, USA. The primary antibody used for aggrecan (1R11) was obtained from (Invitrogen-Molecular Probes, Grand Island, NY, USA). The cascade of antigen-antibody complexes formed was identified by the peroxidase anti-peroxidase method (VECTASTAIN Elite ABC Kit-mouse Vector Laboratories, Burlingame, CA, USA), catalyzed by alkaline phosphatase and, was converted into a detectable signal in the presence of a chromogenic substrate like pNNP. Absorbance was then measured from the resulting calorimetric reactions at a wavelength of 405 nm. The amount of specific antigen present in the samples was then determined by the linear relationship (standard curve not available in the given article) established between different antibody concentrations and the corresponding absorbance measurements.

### 4.7. Statistical Analysis

Quantitative analyses of fibrochondrocyte proliferation and functionality were analyzed using two-way analysis of variance (ANOVA) with statistical significance set at *p* < 0.05. Values are expressed as mean ± SD (*n* = 6).

## 5. Conclusions

TiO_2_ nanoparticle thin films were assembled through LbL nanoassembly. Temporomandibular joint fibrochondrocytes thrived on these substrates and maintained their phenotype and functionality on TiO_2_ nanofilms. An increase in the number TiO_2_ nanofilm bilayers was shown to enhanced fibrochondrocyte cell behavior. While this study focused on fibrochondrocytes, we believe other cell types, such as chondroblasts, osteoblasts and mesenchymal stem cells will also grow and maintain functionality on these nanoparticle nanofilms.
